# Mode of reduction in the number of pharyngeal segments within the sarcopterygians

**DOI:** 10.1186/s40851-016-0043-6

**Published:** 2016-03-21

**Authors:** Victoria Shone, Silvan Oulion, Didier Casane, Patrick Laurenti, Anthony Graham

**Affiliations:** Department of Developmental Neurobiology, Kings College London, London, SE1 1UL UK; Laboratoire Évolution, génomes, comportement, écologie, CNRS université Paris-Sud UMR 9191, IRD UMR 247, Avenue de la Terrasse, bâtiment 13, boîte postale 1, 91198 Gif-sur-Yvette, France

**Keywords:** Pharyngeal, Segmentation, Sarcopterygians, Tetrapods, Hoxb1, Endoderm

## Abstract

**Background:**

Pharyngeal segmentation is a defining feature of vertebrate embryos and is apparent as a series of bulges found on the lateral surface of the embryonic head, the pharyngeal arches. The ancestral condition for gnathostomes is to have seven pharyngeal segments: jaw, hyoid, and five posterior branchial arches. However, within the sarcopterygians, the pharyngeal region has undergone extensive remodelling that resulted in a reduction in the number of pharyngeal segments, such that amniotes have only five pharyngeal arches. The aim of this study is to probe the developmental basis of this loss of pharyngeal segments.

**Results:**

We have therefore compared the development of the pharyngeal arches in an amniote, the chick, which has five segments, with those of a chondrichthyan, the catshark, which has seven segments. We have analysed the early phase of pharyngeal segmentation and we find that in both the most anterior segments form first with the posterior segments being added sequentially. We also documented the patterns of innervation of the pharynx in several vertebrates and note that the three most anterior segments receive distinct innervation: the first arch being innervated by the Vth nerve, the second by the VIIth and the third by the IXth. Finally, we have analysed Hox gene expression, and show that the anterior limit of *Hoxa2* aligns with the second pouch and arch in both chick and catshark, while *Hoxa3* is transiently associated with the third arch and pouch. Surprisingly, we have found that *Hoxb1* expression is spatially and temporally dynamic and that it is always associated with the last most recently formed pouch and that this domains moves caudally as additional pouches are generated.

**Conclusion:**

We propose that the first three pharyngeal segments are homologous, as is the posterior limit of the pharynx, and that the loss of segments occurred between these two points. We suggest that this loss results from a curtailment of the posterior expansion of the pharyngeal endoderm in amniotes at relatively earlier time point, and thus the generation of fewer segments.

## Background

Pharyngeal segmentation is a characteristic of all vertebrates. During development it is first seen in the appearance of a series of bulges on the lateral surface of the head, the pharyngeal arches [1]. These primordia consist of epithelia with a mesenchymal filling. Externally, the epithelium is derived from the ectoderm and internally from the endoderm, while the mesenchyme consists of a core of mesoderm surrounded by neural crest cells. Between the arches the ectoderm and the endoderm contact each other, and it is this apposition between these tissues that defines the anterior and posterior margins of each of the pharyngeal arches [1].

A general feature of gnathostomes is to have seven pharyngeal segments; jaw, hyoid, and five posterior branchial (gill-bearing) arches [2]. This arrangement represents the plesiomorphic state as this situation is observed in many chondrichthyans and all actinopterygians, and this arrangement is also seen in sarcopterygians, such as the coelacanth *Latimeria* and the lungfishes, the sister group to the tetrapods [3–6]. However, extant tetrapods show a reduced number of pharyngeal segments. Thus, larval amphibians have six pharyngeal segments, while amniote embryos have only five. Moreover, there has also been a loss of pharyngeal segmentation in the adult form as a result of the remodelling of the pharynx, which occurs during metamorphosis in amphibians and embryogenesis in amniotes. The reduction in the number of pharyngeal segments and the loss of explicit segmentation in adult tetrapods clearly reflects the shift from respiration in water via gills to air breathing using lungs.

These alterations to the pharyngeal region that occurred with the evolution of the tetrapods are obviously underpinned by changes to the developmental programme. The loss of overt pharyngeal segmentation is due to the overgrowth of the second arch to cover the more posterior arches, followed by the fusion of the caudal edge of the second arch to the subjacent tissue which results in the internalisation of the posterior arches [7]. However, it is less clear how the reduction in the number of pharyngeal segments has been achieved, although this is likely to involve changes to the early organisation of the pharyngeal endoderm.

The segmentation of the endoderm, which results in the formation of the pharyngeal pouches, is central to the development of the pharyngeal arches [8]. The pharyngeal pouches form at distinct positions along the anteroposterior axis. The two most anterior pouches form first with the more posterior pouches forming subsequently and sequentially [9, 10]. The pouches grow to contact the overlying ectoderm, which invaginates to meet them, generating the pharyngeal clefts, and thus neural crest cells and mesoderm migrate into these preformed units. Significantly, in mutants in which the endoderm fails to segment, the pharyngeal arches fail to form [11, 12]. Consequently, a reduction in the number of pharyngeal segments in sarcopterygians must have involved alterations to the development of the pharyngeal pouches.

To begin to address the route through which the number of pharyngeal segments has been reduced, we have compared the development of the pharyngeal arches in the chick (*Gallus gallus*), an amniote, which has five segments, to those in the catshark (*Scyliorhinus canicula*), a chondrichthyan, which has seven segments. We have analysed the formation of pharyngeal pouches, patterns of innervation of the pharyngeal arches, and the expression of *Hox* genes, which are markers of axial identity in the pharynx, and in particular how these relate to the endodermal pouches. Our data support the view that the three most anterior pharyngeal segments are homologous between different vertebrate classes, and that the reduction in the number of segments must have been achieved by a loss of those lying more caudally. Notably, we find that in both chick and catshark embryos *Hoxb1* expression marks the posterior limit of the pharynx. However, this expression pattern is spatially and temporally dynamic, with *Hoxb1* labelling the posterior limit of the pharynx at early and late stages irrespective of the number of segments that have been generated. Thus we conclude, that the posterior limit of the pharynx is likely to be homologous across the gnathostomes and that the reduction in the number of pharyngeal segments that accompanied the evolution of the tetrapods will have involved a heterochronic shift such that the posterior expansion of the pharynx was curtailed at a relatively earlier time point and thus less segments were generated.

.

## Materials and methods

### Embryo collection

Fertile hen’s eggs were incubated at 38 °C to the required stages (HH st) [13] and the embryos were fixed in 4 % PFA. *Scyliorhinus canicula* embryos were taken from the egg cases, anaesthetized (MS222), staged [14] and fixed in 4%PFA. Lamprey embryos were kindly donated by Dr Sebastian Shimeld, University of Oxford.

### Immunohistochemistry

Previously fixed embryos were washed three times 30 min in PBS/1 % TritonX-100 (PBSTx) before being washed in a blocking solution of 10 % goat serum in PBSTx twice for one hour at room temperature. The relevant primary antibody was diluted in the blocking solution with 0.02 % sodium azide and the embryos incubated at 4 °C for 1–2 weeks. Embryos were then rinsed in blocking solution and washed three times for one hour in blocking solution before adding the secondary antibody diluted in blocking solution with 0.02 % sodium azide. This was incubated at 4 °C for 1–2 weeks. The primary antibodies used were rabbit anti-laminin at 1:100 (Sigma); mouse anti- NFM 1:10000 (Zymed). Secondary antibody was Alexa 488-conjugated goat anti-mouse IgG, and Alexa 568 goat anti-rabbit IgG, both used at 1:1000 (Molecular Probes). For sectioning, embryos were washed into PBS, embedded in gelatin, fixed, and vibratomed at 50 μm slices.

### In situ hybridisation

Previously fixed embryos were washed twice in PBST (PBS + 0.1 % Tween-20), dehydrated through a methanol series in PBST, bleached with 6 % H_2_O_2_ in methanol for 1 h and then rehydrated. Embryos were then treated with 10 μg/ml Proteinase K in PBST for 20 min, postfixed with 4 % PFA/0.1 % gluteraldehyde and then washed twice in PBST. The embryos were incubated at 70 °C in hybridisation buffer (50 % formamide, 1.3X SSC, 5 mM EDTA, 50 μg/ml tRNA, 100 μg/ml heparin, 0.2 % Tween-20, 0.5 % CHAPS) for 1 h, followed by overnight at 70 °C in digoxigenin-labelled riboprobes. Embryos were washed four times with hybridisation buffer at 70 °C, then three times 30 min with MABT (100 mM maleic acid, 150 mM NaCl, 1 % Tween-20; pH7.5), and blocked with 2 % BBR (Roche Diagnostics)/20 % goat serum/MABT and incubated overnight in anti-DIG-AP antibody (Roche) diluted 1:2000 in the same block. Embryos were washed extensively with MABT and the alkaline phosphatase activity detected using NBT and BCIP in NTMT. The reaction was stopped by washing in MABT and fixing in 4 % PFA.

## Results

### Sequential generation of pharyngeal segments

**Fig. 1:**
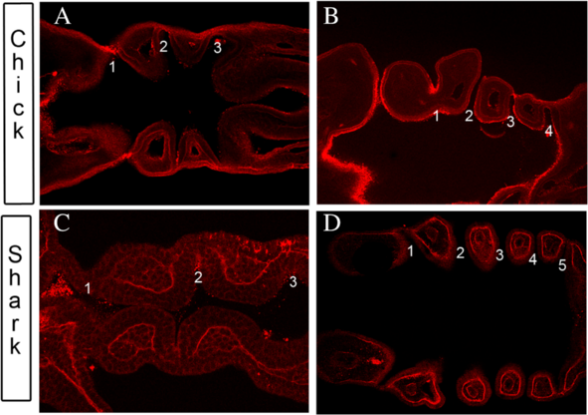
Overview of pharyngeal segmentation in Chick (*Gallus gallus*) and catshark (*Scyliorhinus canicula*) embryos. Longitudinal sections through the pharyngeal region of whole mount anti-laminin immunostained chick (**a, b**) and catshark (**c, d**) embryos during the period of segmentation. **a** Section through a stage 14 chick embryo, at which point the first three pharyngeal pouches have formed. The contact between the pouches and the endoderm can be clearly seen. **b** Section through a stage 19 embryo, by which time all four pharyngeal pouches have formed. It is now quite clear that the different pouches have assumed different morphologies. The first pouch has receded from the ectoderm and mesenchyme lies between, while the second pouch has broken through to create an external opening. The interface between pouch 3 and the ectoderm is thinning at this point while pouch 4 is still abutting the ectoderm. **c** Section through a stage 19 catshark embryo, at which point the first three pouches have formed and are in contact with the ectoderm. **d** Section through a stage 22 catshark embryo which has formed 5 pouches. At this stage pouches 1 to 4 have broken through externally while the fifth pouch is just beginning to do so

We used anti-laminin immunostaining to conduct a general analysis of the formation of the pharyngeal pouches in both chick and catshark embryos. The pharyngeal segments form first anteriorly and then subsequently posteriorly, in both species (Fig. 1). The first two pharyngeal pouches in chick form at stage 13 [15], and by stage 14 (Fig. 1a) these have fully formed and are in intimate contact with the ectoderm. At this stage it is also clear that the third pharyngeal pouch is forming and has contacted the ectoderm, defining the posterior limit of the third arch. By stage 19 of chick development, the fourth, and last pouch, has formed and four distinct pharyngeal segments are apparent (Fig. 1b). The final segment, the sixth arch will soon form between the fourth pouch and the posterior limit of the pharynx; even though this is numerically the fifth arch, this is termed the sixth due to the long held, but erroneous, belief that a transient fifth arch formed between this segment and the fourth [16]. By this stage, the first pouch has now receded from the ectoderm and mesenchyme lies between these tissues, while the second pouch has broken through and created an external opening. The posterior pouches, 3 and 4, remain in contact with the ectoderm. Thus the anterior and posterior pouches interface with the ectoderm in different ways and are morphologically distinct. In catshark, the anterior pharyngeal segments are also delineated first and by stage 19 the three most anterior pouches have formed and are in contact with the ectoderm (Fig. 1c). By stage 22 of catshark development, pouches 4 and 5 have formed, yielding five distinct arches. The anterior pouches have also matured and these have pushed through and created external pharyngeal openings, with only pouch 5 not having broken through, as the ectoderm and endoderm remain opposed to each other at this position (Fig. 1d). In contrast to the chick, however, the relationships between the pouch endoderm and the ectoderm are relatively consistent across the pouches and these share a similar morphology.

### Analysis of pharyngeal innervation further identifies the most posterior segments as being reduced in number

**Fig. 2:**
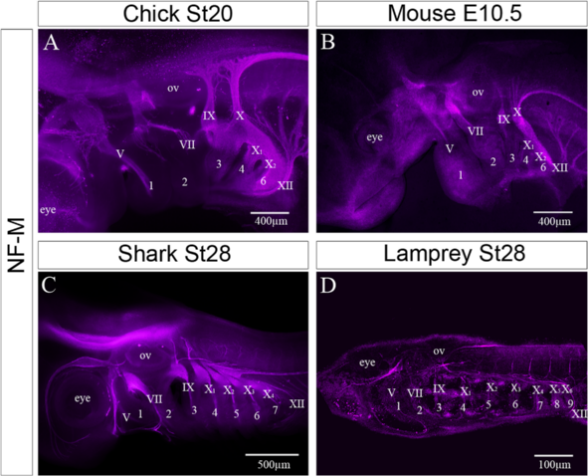
Comparative analysis of pharyngeal innervation across the verterbates. Patterns of innervation of the developing pharyngeal arches in different vertebrates at the point at which the full complement of pharyngeal segments have been generated. The embryos were wholemount immunostained with and anti-neurofilament antibody and the either bisected and photographed or photographed whole. **a** Chick **b** Mouse **c** Catshark **d** Lamprey. The cranial nerves are labelled with roman numerals. V – trigeminal, VII – facial, IX – glossopharyngeal, X – vagus, XII – hypoglossal. The pharyngeal arch are numbered with Arabic numerals. The position of the eye is indicated as is the position of the otic vesicle (OV). It is clear that in all embryos there is distinct innervation of the first arch by the Vth nerves, of the second arch by the VIIth nerve and of the third by the IXth nerve. In all embryos the XIIth nerve skirts the caudal edge of the pharynx. It is also apparent that there are a variable number of branches of the Xth nerve innervating the variable number of posterior segments

An examination of the patterns of cranial nerve innervation of the pharynx in different vertebrate embryos can prove useful in focussing on which specific arches are likely to have been lost. We have therefore documented the patterns of innervation of the pharyngeal segments in two amniotes, chick and mouse, which have five segments, in a chondrichthyan, the catshark, which has seven segments, and in the lamprey which has nine. Wholemount immunofluorescence with an anti-neurofilament antibody is shown in Fig. 2. It is readily apparent that clear and distinct innervation arches 1, 2 and 3 can be seen across all species; i.e., innervation of the first segment by the Vth nerve, the second by the VIIth nerve, and the third by the IXth nerve. It is also clear that the posterior limit of the pharynx is skirted by the XIIth nerve in all. Finally, the variable number of posterior segments are innervated by a variable number of branches of the Xth nerve. This suggests that the anterior three segments are homologous across the vertebrates, and that there has been a loss of segments posterior of the third.

### *HOX* gene expression boundaries and their relationship to the pharyngeal arches and pouches in chick embryos

To ascertain which pharyngeal segments/pouches have been lost from extant tetrapods, we need to be able to align the reduced number of posterior pouches of an amniote, in this case the chick, with those of the catshark, which represents the more basal condition. With regard to this issue, *Hox* genes are useful as they can be used as markers of axial identity. Although the expression patterns of *Hox* genes in the pharyngeal arches have been well documented in amniotes, these studies have largely reported expression within the mesenchyme of the arches and they have often not explicitly analysed the limits of expression of these genes within the pharyngeal endoderm [17, 18].

**Fig. 3:**
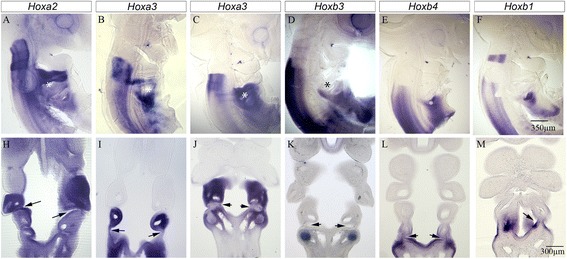
Hox gene expression boundaries and their relationship to the pharyngeal arches and pouches in the chick embryo. Patterns of expression of *Hox* genes in the developing pharynx at stage 20 (**a**, **b**, **d**-**g**, **h**, **i**, **k-** **n**) when all of the pharyngeal segments have formed. This figure additionally shows the expression of *Hoxa3* at stage 17 (**b**, **i**). The expression patterns of these genes are shown in bisected embryos (**a** – **g**) and in section (**h**-**n**). The expression pattern of *Hoxa2* is shown in (**a**, **h**) and this gene can be seen to be expressed in the mesenchyme of the second and more posterior arches and in the endoderm of the second pouch and posterior of that. The position of the second pouch is marked by an asterisk in (**a**) and an arrow in (**h**). The expression of *Hoxa3* is shown at stage 17 (**b**, **i**) and at stage 20 (**c**, **j**). In these panels the position of the third pouch is marked by an asterisk in (**b**, **c**) and and arrow in (**i**, **j**). The expression pattern of *Hoxa3* in the mesenchyme does not change between these stages and can be seen to be expressed in the third arch and more posterior. However, while *Hoxa3* can be seen to be expressed in the third pharyngeal pouch at stage 17 (**i**), expression is lost from here at stage 20 (**j**). The expression of *Hoxb3* is shown in (**d**, **k**) and this gene can be seen to be expressed in the mesenchyme of the fourth arch but its expression is noticeably absent from the pharyngeal endoderm (**k**). The position of the third pouch is marked by an asterisk in (**d**) and by an arrow in (**k**). The expression of *Hoxb4* is shown in (**e, l**) and this gene can be seen to be expressed is not expressed in the mesenchyme of any of the arches, nor in any of the pharyngeal pouches but is expressed in the most posterior pharyngeal endoderm and the ectoderm lying posterior of the pharynx. The position of the fourth pouch is marked by an asterisk in (**e**) and by an arrow in (**l**). The expression of *Hoxb5* is shown in (**f**, **m**) and it can be seen that this gene is not expressed in the pharyngeal region. *Hoxb1* expression is shown in (**g**, **n**) and at stage 20 it is clear that the expression of this gene is restricted to the last formed, fourth, pharyngeal pouch, marked by an asterisk in (**g**) and an arrow in (**n**)

We therefore analysed the expression patterns of *Hox1-5* gene paralogues, as these are known to be expressed in the pharyngeal region [17, 18]. As anticipated, the different paralogues labelled different arches (Fig. 3a–g). We then sectioned these embryos to determine the expression domain of these genes within the different populations that constitute the arches, and in particular in the endoderm, at stages when all four pharyngeal pouches had formed, stage 20. We note that the first pharyngeal pouch is not associated with the expression of any *Hox* genes. However, we found that *Hoxa2* was expressed in the second arch mesenchyme and the endoderm posteriorly from the second pouch (Fig. 3h) while *Hoxa3* was expressed in the third arch mesenchyme and the third pouch endoderm at stage 17 (Fig. 3i). However, although expression of *Hoxa3* persists in the third arch mesenchyme the expression in the third pouch was subsequently lost (Fig. 3j). This is in keeping with what has been observed in the mouse [19]. Contrastingly, we found that *Hoxb3* expression was absent from the endoderm, but that this gene was expressed in the mesenchymal cells that will populate the fourth arch (Fig. 3k). We found *Hoxb4* expression was not associated with any of the pharyngeal pouches but was expressed in the most posterior pharyngeal endoderm and the ectoderm lying posterior of the pharynx (Fig. 3l). We also found that *Hoxb5* was not expressed in the developing pharynx (Fig. 3f, m). However, we did find that the expression of *Hoxb1* was localised to the fourth pouch, the last formed, and at this stage the expression in the mesoderm of the second arch was no longer evident (Fig. 3g, n). This would suggest that *Hoxb1* may be useful as a specific marker of the fourth and/or last pouch formed.

### *Hox* gene expression boundaries and their relationship to the pharyngeal pouches in catshark embryos

The overall relationship between the pharyngeal arches and *Hox* gene expression in catshark (*Scyliorhinus canicula*) has been described previously and it is broadly similar to the situation observed in amniotes, with *Hox* genes of paralogous groups (PG) 1–4 being expressed in the pharyngeal region, and *Hox* gene of paralogous groups (PG) 5–8 not being expressed [20]. There were, however, also some important detailed differences, such as the fact that *Hoxd3* expression had shifted posteriorly and was associated with the fourth arch, and that this segment additionally expressed *Hoxd1*, *d2* and *d4*. The precise relationship between the expression of *Hox* genes and the pharyngeal pouches has not been scrutinised in any chondrichthyan, but such analysis is vital if we wish to align the pharyngeal pouches across the gnathostomes. The key questions are: Does *Hoxa2* expression align with the second pouch in both amniotes and a chondrichthyan, and does *Hoxb1* expression which highlights the fourth and last pouch of amniotes align with the fourth pouch in a chondrichthyan, or is this gene expressed by multiple posterior pouches, or indeed only in the sixth and last formed pouch?

**Fig. 4:**
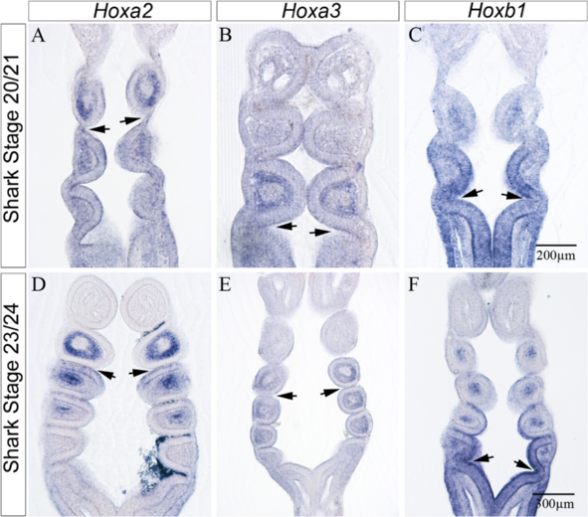
Hox gene expression boundaries and their relationship to the pharyngeal pouches in *Scyliorhinus canicula.* Expression patterns of *Hoxa2*, *Hoxa3* and *Hoxb1* in early (**a**-**c**), stage 20/21which have formed 3 to 4 pharyngeal arches, and later (**d**-**f**) embryos, stage 23/24 which have formed 5 to 6 pharyngeal arches. At both early and late stages *Hoxa2* is expressed in the mesenchyme of the second and more posterior arches and in the endoderm caudal of the second pouch, marked by an arrow (**a**, **c**). *Hoxa3* shows expression in the mesenchyme of the third and more posterior arches at both early and late stages (**b**, **e**) but only weak (**b**) to no expression in the pharyngeal endoderm. The position of the third pouch (**b**, **e**) is indicated by the arrow. *Hoxb1* shows prominent expression in fourth pharyngeal pouch at early stages, marked by an arrow (**c**) and in the fifth pouch at later stages, marked by an arrow (**f**)

Our analysis of the chick highlighted the fact that the different pouches were primarily marked by the expression of *Hoxa2*, *Hoxa3* and *Hoxb1* and we therefore sectioned wholemount in situ hybridisations of catshark embryos processed for these genes. We found that *Hoxa2* expression was present in the mesenchyme of the second and more posterior arches and the endoderm posterior of the second pouch between stages 20 to 24 (Fig. 4a, d). Contrastingly, *Hoxa3* expression was found in the mesenchyme of arch 3 at stages 21 to 24, but that expression in the endoderm of the third pouch was weak at stage 21 and absent at later stages (Fig. 4b, e). Finally, the expression of *Hoxb1* was found in the third pouch at stage 20, and at stage 24 in the fifth pouch (Fig. 4c, f). Thus *Hoxb1* expression is associated with different pharyngeal pouches at different developmental stages.

### Transient Hoxb1 expression marks the caudal limit of the pharynx

**Fig. 5:**
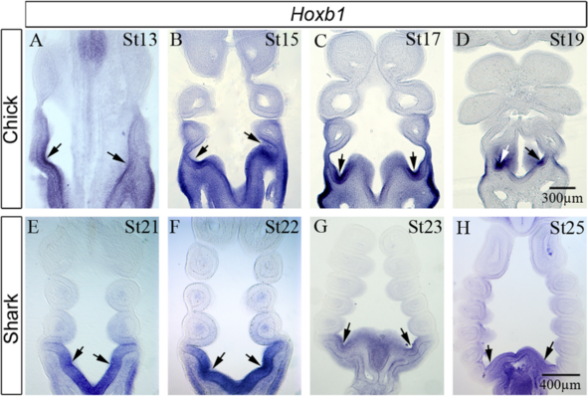
Hoxb1 expression is spatially and temporally associated with the last formed pharyngeal pouch in both chick and catshark. Spatially and temporally dynamic expression of *Hoxb1* is associated with the last formed pharyngeal pouch in both chick (**a**-**d**) and catshark (**e**-**h**) embryos. At stage 13 in the chick *Hoxb1* expression is associated with the newly formed second pouch (**a**), while at stage 15 the expression of this gene is found in the newly formed third pouch (**b**). From stage 17 onwards, *Hoxb1* expression is associated with the fourth pouch (**c**, **d**). In catshark embryos a similar spatially and temporally dynamic association between the expression of *Hoxb1* and the last formed pouch is also seen, Thus while at stage 21, the expression of this gene is found at the fourth pouch (**e**), by stage 22 it is located to the fifth pouch (**f**) and by stage 23 and later with the sixth pouch (**g**, **h**). In all panels the position of the last formed pouch is highlighted by an arrow

The association between the expression of *Hoxb1* with different pouches at different stages of development in *Scyliorhinus* was surprising, and we therefore set out to determine if this was a general feature of pharyngeal development in both chick and catshark. The first two pharyngeal pouches form in the chick at stage 13 and we found that even at this early time point *Hoxb1* expression was already established in the most caudal pouch, pouch 2 (Fig. 5a). By stage 15 the third pouch had formed and we noted that *Hoxb1* expression was now associated with that newly formed pouch (Fig. 5b). Finally, we found that by the time all four pouches had formed, *Hoxb1* expression had shifted to this last pouch (Fig. 5c, d). Intriguingly, a more detailed examination of the expression profile of this gene in catshark revealed a similar situation. At stage 21 when four pouches had formed *Hoxb1* expression was associated with the newly formed fourth pouch (Fig. 5e) while at stage 22 when the fifth pouch was forming, *Hoxb1* expression had shifted to this pouch (Fig. 5f). Finally, *Hoxb1* expression shifted to the lastly formed sixth pouch from stage 23 onwards Fig. 5g, h).

## Discussion

In the present study, we have attempted to address the route through which the reduction in the number of pharyngeal segments that occurred within the sarcopterygians, and which is evident in extant tetrapod embryos. To do this, we compared the development of the pharyngeal segments in an amniote, the chick, with that in a chondrichthyan, the catshark. We show that in both the overall trend in the formation of the pharyngeal pouches is similar, with the most anterior forming first and then the posterior segments forming sequentially. We further analysed the patterns of innervation and the expression profiles of *Hox* genes in the forming arches, and in particular how these relate to the pharyngeal pouches, which dictate the number of segments formed. Our results show support for the first three anterior segments being homologous across the gnathostomes. Each of these had a distinct innervation, and both the mesenchyme of these arches and the corresponding pouches expressed the same repertoire of *Hox* genes in both chick and catshark. To gain insights into how the posterior pouches align between chick and catshark, we analysed the expression of *Hoxb1*, which is expressed in the most posterior pouch in amniotes. Somewhat unexpectedly, we found that *Hoxb1* exhibits a dynamic expression profile during the formation of the pharyngeal segments, with the expression domain of this gene progressively moving caudally such that at any given stages it is expressed in the last formed pouch. This leads us to suggest that the posterior limit of the pharynx is homologous between chick and catshark, and that a reduction in the number of pharyngeal segments has been achieved by the earlier termination of the caudal expansion of the pharyngeal endoderm. Thus the caudal limit of the pharynx is established at a relatively more anterior position and therefore fewer pharyngeal pouches form and correspondingly fewer pharyngeal segments.

**Fig. 6:**
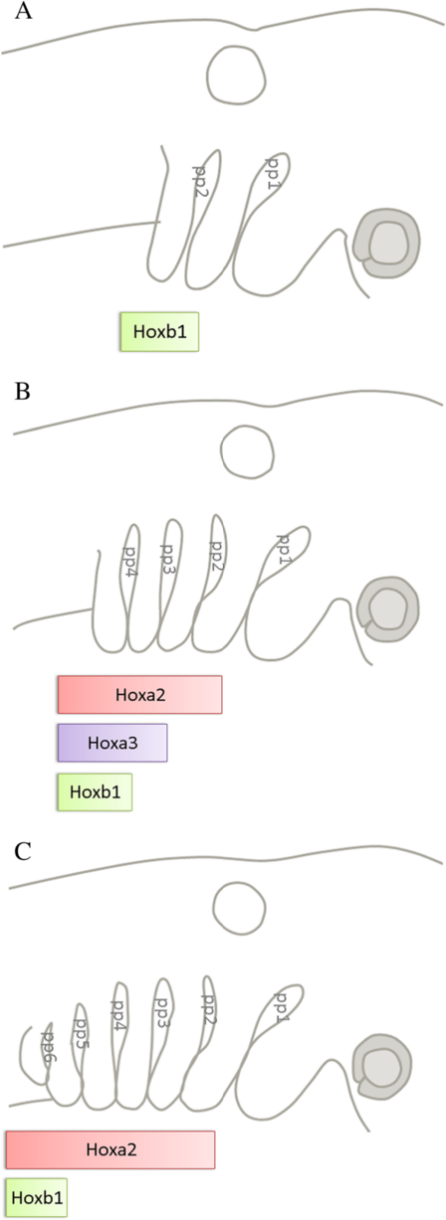
A conserved pattern of Hox gene expression aligns with the pharyngeal pouches. Schematic representation of *Hox* gene expression in the pharyngeal pouches of a generalised gnathostome throughout pharyngeal development. **a** Initial pharyngeal pouch formation begins with the simultaneous budding off the pharyngeal endoderm of the first and second pharyngeal pouches. *Hoxb1* aligns with the second, most posterior pharyngeal pouch. **b** At slightly older stages, four pharyngeal pouches present. *Hoxa2* is now expressed with an anterior limit in the second pouch, *Hoxa3* with an anterior limit at the third pouch. *Hoxb1* no longer has an anterior limit at the second pouch but is now present in the most posterior pouch, which at this stage is the fourth. **c** Once all pouches have developed, the anterior limit of *Hoxa2* expression still aligns with the second pouch while *Hoxa3* expression is no longer seen in the endoderm. *Hoxb1* expression no longer aligns with the fourth pouch but rather with the most posterior pouch, which at this stage is the sixth pouch. This represents a conserved dynamic expression pattern of *Hoxb1* in the most posterior pouch present of gnathostomes at any given time point during pharyngeal pouch development irrespective of the number of pouches that have formed at that time. ;(pp = pharyngeal pouch)

From our analysis we can begin to understand which of the posterior, or branchial, segments have been lost. Our results clearly indicate that the most anterior of the branchial series, the third pharyngeal segment is homologous across the gnathostomes and is retained in amniotes. In lamprey, catshark, chick and mouse, this segment is distinctly innervated by the IXth nerve. Furthermore, we show that the expression of *Hoxa3*, in chick and catshark in both the mesenchyme and the corresponding pouch endoderm is also a conserved feature of the third segment; although, the expression of this gene is lost as development progresses. However, it is less clear which of the more posterior arches have been retained and which have been lost. In part this is due to the fact that the expression patterns of *Hox* PG 3 and 4 do not simply align with distinct arches or pouches in either chick or catshark embryos. Finally, our study suggests that the posterior limit of the pharynx may be homologous, in that it is defined by the expression of *Hoxb1* in both species (Fig. 6). Indeed, we find that the anterior limit of Hoxb1 expression initially marks pouch 2, i.e. the anterior limit of the third arch and thus the anterior limit of the branchial apparatus in anamniotes. This is consistent with the view that the third pharyngeal arch is homologous across the gnathostomes. Additionally, we show here that in both chick and catshark the limit of expression of this gene shifts posteriorly as development progresses and thus it labels sequentially the caudal most brachial pouch. Taken together our observations indicate that the four most anterior arches were retained in amniotes but that the two segments that form subsequently were lost between the fourth arch and the caudal limit of the pharynx.

While our work gives an insight into the possible mechanism that underpinning the reduction in arch number that occurred within the sarcopterygians, the morphological data form fossils also provides a very useful complement that give us insights into when the reduction in the number of pharyngeal segments started [21]. Thus while extant and fossil coelacanths have five branchial arches, it has been reported that *Gogonasus* and *Eusthenopteron*, which are tetrapodomorph fish, have lost the fifth arch. Thus it has been suggested that the absence of the fifth branchial arch is a derived feature of advanced sarcopterygians.

Importantly, other studies suggest that the mode of arch reduction that we have identified, in which deletion occurs between a *Hox1*-defined posterior limit of the pharynx and more anterior segments, is likely to extend beyond events within the sarcopterygians. An analysis of Hox gene expression in the lamprey, *Lethenteron japonicum*, found that the *Hox1* gene, *LjHox1w*, in this this species also marks the caudal limit of the pharynx, although it is unclear whether *LfHox1w* displays a similar temporally and spatially dynamic pattern of expression [22]. Perhaps even more significantly, an analysis of pharyngeal gill slit formation in a hemichordate, *Saccoglossus kowalevskii*, which concludes that this process is homologous to the formation of the pharyngeal segments of vertebrates, also reported spatially and temporally dynamic expression of *Hox1* [23]. In that study, the authors noted that at *Hox1* expression was restricted to the posterior boundary of the pharyngeal endoderm at the three gill-slit stage and that it was still restricted to the posterior boundary of the pharyngeal endoderm at the four gill-slit stage. Collectively, our work and those of others therefore suggest that a possible role for *Hox1* genes in defining the caudal limit of the pharynx extends beyond the vertebrates, and is likely to have evolved much earlier with the emergence of the deuterostomes.

This is also significant in that it could help us understand how the number of pharyngeal segments has been modified across the deuterostomes. Thus while enteropneust hemichordates and cephalochordates have numerous pharyngeal gill slits [24, 25], which extend significantly along the length of the body, those in vertebrates are fewer in number and are focussed just caudal of the mouth. Thus, the number of pharyngeal segments could be decreased by terminating the posterior expansion of the pharynx prematurely and so decreasing the distance between the *Hox1* expression domain and the last formed anterior segment. Correspondingly, an increase in the number of pharyngeal segments, Thus the number of pharyngeal segments could be increased, such as is seen in the extinct jawless vertebrate *Endeiolepis* which had up to thirty pairs of gill slits [26], by allowing the posterior of the pharynx to extend for a relatively longer time and thus increase the region between the *Hox1* expression limit and the anterior.

## Conclusions

Our results support the view that the three most anterior pharyngeal segments are conserved across the vertebrates and that the caudal limit of the pharynx is also conserved. Thus, within the sarcopterygians, the segments that were lost were those form the “branchial”/posterior region which lie between the third arch and the caudal limit. Furthermore, our results would suggest the reduction in the number of pharyngeal segments was achieved as a result of the premature termination of the posterior extension of the pharyngeal endoderm which in turn would result in the generation of fewer segments. Finally, we suggest that such a mechanism may also account for the variability in the number of pharyngeal segments seen across the vertebrates and other deuterostomes.
